# Features, Causes and Consequences of Splanchnic Sequestration of Amino Acid in Old Rats

**DOI:** 10.1371/journal.pone.0027002

**Published:** 2011-11-08

**Authors:** Marion Jourdan, Nicolaas E. P. Deutz, Luc Cynober, Christian Aussel

**Affiliations:** 1 Laboratory of Biological Nutrition EA 4466, Paris Descartes University, Paris, France; 2 Department of Surgery, Maastricht University, Maastricht, The Netherlands; 3 Clinical Chemistry, Cochin and Hotel-Dieu Hospitals (APHP), Paris, France; 4 Nutrition Unit, PUI, Henri-Mondor Hospital (APHP), Créteil, France; Institut Pluridisciplinaire Hubert Curien, France

## Abstract

**Rationale:**

In elderly subjects, splanchnic extraction of amino acids (AA) increases during meals in a process known as splanchnic sequestration of amino acids (SSAA). This process potentially contributes to the age-related progressive decline in muscle mass *via* reduced peripheral availability of dietary AA. SSAA mechanisms are unknown but may involve an increased net utilization of ingested AA in the splanchnic area.

**Objectives:**

Using stable isotope methodology in fed adult and old rats to provide insight into age-related SSAA using three hypotheses: 1) an increase in protein synthesis in the gut and/or the liver, 2) an increase in AA oxidation related to an increased ureagenesis, and 3) Kupffer cell (KC) activation consequently to age-related low-grade inflammation.

**Findings:**

Splanchnic extraction of Leu (SPELeu) was doubled in old rats compared to adult rats and was not changed after KC inactivation. No age-related effects on gut and liver protein synthesis were observed, but urea synthesis was lower in old rats and negatively correlated to liver Arg utilization. Net whole-body protein synthesis and arterial AA levels were lower in old rats and correlated negatively with SPELeu.

**Conclusion:**

SSAA is not the consequence of age-related alterations in ureagenesis, gut or liver protein synthesis or of KC activity. However, SSAA may be related to reduced net whole-body protein synthesis and consequently to the reduced lean body mass that occurs during aging.

## Introduction

Aging is characterized by a progressive decline in muscle protein stores [1]. This could be the consequence of a number of factors, including deregulation of protein turnover. Studies conducted in patients in the postabsorptive state did not show any effect of aging on protein kinetics when the results were adjusted for lean body mass [2], [3]. However, studies conducted in the fed state showed that aging results in a blunted stimulation of muscle protein synthesis [4]–[Bibr pone.0027002-Dardevet2] [6] that may be related to the progressive loss of muscle mass characterizing sarcopenia [7], [8]. Furthermore, the contribution of muscle to whole-body protein turnover appears diminished in elderly subjects [9]. Therefore, the inability of elderly people to maintain body protein stores could be attributed to an impaired anabolic response to meals.

The systemic availability of dietary amino acids is a key determinant of protein synthesis [10]. Splanchnic tissues (i.e. mainly gut and liver) may play an important role in this process as these tissues are responsible for the absorption of dietary amino acids and their subsequent release to peripheral tissues (such as muscle). Importantly, first-pass splanchnic extraction of dietary leucine (Leu) [11] and phenylalanine (Phe) [12] increases with age and defines age-related splanchnic sequestration of amino acids (SSAA).

However, there are still no data available from experimental *in vivo* models, and thus the mechanisms underlying SSAA remain unknown. Several hypotheses can be raised. The age-related increase in splanchnic bed amino acid utilization may be explained by an increase in gut and/or liver protein synthesis rates and/or an increase in dietary amino acid oxidation which relates to urea production. An intriguing feature of the splanchnic sequestration of leucine is that this branched chain amino acid (BCAA) is poorly metabolized by hepatocytes as these cells exhibit low levels of the necessary BCAA transaminase [13]. However, the BCAA transaminase activity of the resident macrophages in the liver (i.e. Kupffer cells; KC) is stimulated when these cells are activated [14]. As aging also is characterized by a low-grade chronic inflammation with elevated IL-6 [15], [16] and CRP [17] serum levels, an attractive hypothesis is that this chronic inflammatory state leads to KC activation that in turn could be responsible for the increased extraction of leucine in the splanchnic area.

The purpose of the present work was to gain more insight into SSAA and its consequences on peripheral amino acid availability and protein metabolism. To test our hypotheses, we conducted experiments in healthy adult and old rats in the fed state, using stable isotopes (Leu, Phe, urea and arginine) to measure splanchnic extraction, liver and gut protein synthesis rate, and urea production. Amino acid levels and whole-body protein kinetics were also measured. We chose to study animals in fed condition for several reasons. First, there is only a limited time window in which anesthetized multicatheterized rats (especially old rats) can be studied in a stable condition, which prevented us from studying the same rats in both fasted and fed conditions. Secondly, splanchnic extraction of dietary amino acids is a phenomenon that takes place in fed state. Third, there is broad consensus that protein synthesis is blunted with aging at the fed state but not at the fasting state [18]–[Bibr pone.0027002-Boirie2] [20]. To study a possible explanation for the phenomenon, we performed an additional experiment in old rats to study the role of KC activity on splanchnic extraction of leucine. For this purpose, KC were inactivated by gadolinium chloride treatment [21], [22].

## Materials and Methods

### Ethics Statement

Concerning the ethical treatment of animals, the law in France is different from other countries. Permission is given to a person to review the ethical compliance of protocol conducted in animals according to the recommendations in the Guide to the French regulations on ethics in experimental research and to allow research to be carried out when these criteria are met. In our Laboratory, Christophe Moinard possesses an authorization (authorization No. 75522) to perform experiments on animals according to the French regulations on ethics in experimental research and thus has the ability to review the ethical aspects of research protocols. Christophe Moinard has reviewed and validated the present study research protocol and allowed the experiment to be carried out. This study was carried out in strict accordance with the recommendations in the Guide to the French regulations on ethics in experimental research. All surgery was performed under isoflurane anesthesia followed by a subcutaneous injection of Buprenorphine to eliminate pain and all efforts were made to minimize suffering.

### Animals

The study used male Sprague-Dawley rats obtained from Charles River (Saint Germain-sur-l'Arbresle, France). During the experiment, the rats were individually housed in standard cages allowing an acclimatization period of fifteen days before the experiment.

The rats were fed a standard chow *ad libitum* (UAR AO4, Villemoisson-sur-Orge, France) and food intake was recorded daily over the acclimatization period. Water was provided *ad libitum.* Rats were subjected to a reversed 12-hour light-dark cycle (light from 8:00 P.M. to 8:00 A.M.) and room temperature was maintained at 22°C.

Experiment 1 used adult (3-month-old; n = 13; A) and old (24-month-old; n = 13; O) rats. In experiment 2, 24-month old rats received an i.v. injection of either gadolinium chloride [GdCl3; 10 mg/kg total body weight (BW); n = 10] or saline (200 µl of NaCl, control group; n = 9) 24 hours prior to the experimental protocol described below. GdCl3 is a recognized toxicant for KC and is commonly used to deplete these cells from the liver. Hardonk et al. [23] reported that GdCl3 not only blocks the phagocytosis of Kupffer cells but also eliminates them, and that the inactivation of Kupffer cells continues from 6 h up to 3 days after a single GdCl3 treatment.

### Experimental protocol used in both experiments

After an overnight fast, the rats were anesthetized by isoflurane inhalation (4.5%, 2 l O_2_/min) followed by a subcutaneous injection of Buprenorphine (Temgesic®; 1 mg/kg BW.) to eliminate pain. Anesthesia was maintained *via* continuous inhalation of isoflurane (2.5%, 1.5 l O_2_/min). During surgery, the rats were kept at a constant 37°C using a temperature controller and heater system (Minerve, Esternay, France). The duodenum (2 catheters), jugular vein (2 catheters), mesenteric vein, abdominal aorta, carotid artery, portal vein, hepatic vein, right renal vein and inferior vena cava were catheterized using a 25-gauge needle held in a silastic tube (Silastic Medical Grade tubing 0.51 mm ID, 0.94 mm OD, Dow Corning Corporation, Medical Products, Midland, MO, USA) and glued with cyanoacrylate (Cyanolit 201) [24].

#### NaCl infusion

Normal saline solution was infused into the jugular vein at rates of 5 ml/h for young adult rats and 10 ml/h for old rats using a low-flow syringe pump (Harvard Apparatus Inc., Les Ulis, France) to compensate for the fluid loss caused by the surgical procedure and to ensure that cross-organ blood flow was maintained throughout the experiment.

#### *Para*-aminohippuric acid infusion protocol

The non-toxic indicator *para*-aminohippuric acid (PAH) (Sigma-Aldrich, L'Isle D'Abeau, France) was used for plasma flow measurements [25]. To allow blood flow measurements across the intestine, liver and hindquarters, a primed (450 µl of 50 mM PAH for adult rats and 900 µl of 50 mM PAH for old rats) continuous infusion of PAH solution was administered via the jugular vein using a low-flow syringe pump (Harvard Apparatus Inc). Infusion rate of a 5 mM PAH solution into the jugular vein was 4.5 ml per hour for young adult rats and 9 ml per hour for old rats. After insertion of the catheters into the mesenteric vein and abdominal aorta, the PAH infusion into the jugular vein was stopped and infusion of a 5 mM PAH solution was started into the mesenteric vein and abdominal aorta at rates of 2.25 ml/h for adult rats and 4.5 ml/h for aged rats [25].

#### Nutrition infusion protocol

The fed state was achieved via continuous infusion of a mixture of free amino acids (Vintène®, Baxter, Derfield, IL, USA, supplemented with glutamine) (Table 1) and maltodextrin (13.3 g/100 ml) into the duodenum. Infusion rate was based on the quantity of nitrogen that the rats would normally have consumed in one hour (27 mg of nitrogen/rat/hour was infused into the duodenum), taking into account the spontaneous protein intake of adult and old rats recorded during the acclimatization period, i.e. 28±1 g and 27±2 g of chow per adult or old animal, respectively.

**Table 1:** Amino acid composition of the mixture infused into the rat duodenum.

	nmol/min
*Essential amino acids*	
Isoleucine (Ile)	1336
Leucine (Leu)	2612
Lysine (Lys)	1712
Methionine (Met)	1174
Phenylalanine (Phe)	1364
Threonine (Thr)	1155
Tryptophan (Trp)	306
Valine (Val)	1496
*Non essential amino acids*	
Alanine (Ala)	3652
Arginine (Arg)	2155
Asparagine (Asn)	564
Cysteine (Cys)	413
Glutamine (Gln)	1421
Glutamate (Glu)	850
Glycine (Gly)	3067
Histidine (His)	645
Ornithine (Orn)	240
Proline (Pro)	2391
Serine (Ser)	714
Tyrosine (Tyr)	55

#### Tracer infusion protocol

A primed-constant infusion of stable isotopes (Mass Trace, Woburn, MA) was given into the jugular vein and the duodenum (see table 2 for details).

**Table 2:** Tracer prime and infusion rates.

Tracer	Abbreviation	Route of continuous infusion	Prime (i.v.) (nmol/rat)	Continuous infusion(nmol/rat/hour)
L-[guanidino-^15^N_2_,5,5,^2^H_2_]-arginine	[^15^N_2_-^2^H_2_]Arg	i.v.	4708	6420
L-[ring-^2^H_5_]-phenylalanine	[^2^H_5_]Phe	i.v.	7629	11600
L-[ring-^2^H_2_]-tyrosine	[^2^H_2_]Tyr	i.v.	1880	3900
L-[1-^13^C]-leucine	[^13^C]Leu	i.v.	5311	8940
L-[5,5,5-^2^H_3_]-leucine	[^2^H_3_]Leu	duodenal	5311	17880
[^13^C]-Urea	[^13^C]-Urea	i.v.	91250	107280

With i.v. = intravenous.

#### Blood sampling and processing

We performed pilot studies (data not shown) to validate showed that steady-state conditions required to measure protein turnover were obtained from 45 min onwards after the start of nutrition infusion. Therefore, time 60 min was chosen for blood sampling. Blood was collected on ice from the carotid artery (arterial blood), portal vein, hepatic vein and inferior vena cava (venous blood) into heparinized cups (Sarstedt, Orsay, France). The amount of blood taken was 500 µl/sample (2.5 ml in total), which equates to approximately 9.3% and 4.5% of the circulating volume in young adult and old rats, respectively (assuming that 8% of total body weight is blood).

For PAH determinations, 50 µl of plasma was added to 250 µl of 6% trichloroacetic acid solution, thoroughly mixed for deproteinization, and then centrifuged.

For determination of amino acid concentrations and tracer-tracee ratio (TTR), 80 µl of plasma was added to 13 mg of 50% solid 5′-sulfosalicylic acid solution, then vortexed, frozen in liquid nitrogen, and stored at −80°C until analysis.

### Body composition evaluation

At T = 60 min, once blood samples had been taken, the rats were killed and dissected to separate and weigh carcass (CW, reflecting fat free mass), skin with attached fat (SKW, reflecting fat mass), abdominal fat, liver, jejunum, ileum and *gastrocnemius* muscle.

### Liver protein content

Frozen liver was pulverized and homogenized in ice-cold 10% trichloroacetic acid (1 mL/100 mg) with an Ultra-Turrax T25 tissue disrupter (Ika Labortechnik, Staufen, Germany). After delipidation with ethanol/ether (1∶1, vol/vol), the total protein precipitate was dissolved in 1 N NaOH (4 mL/100 mg tissue) for 12 hours at 40°C. Total protein content was then assayed according to the method of Gornall [26].

### Sample analysis

Plasma PAH was determined spectrophotometrically on a Cobas Mira S system (Roche Diagnostica, Hoffman La Roche, Basel, Switzerland) by a standard enzymatic method [27]. Plasma amino acid and urea concentrations and TTR were measured using a fully automated LC-MS system (Thermoquest LCQ, Veenendaal, The Netherlands) [28] after precolumn derivatization with FMOC [29].

### Calculations

#### Whole body rate of appearance

Formulae were derived from metabolic studies using radioactive tracers and corrected for the contribution of lower isotopomers as described by Rosenblatt et al. [30]. Whole body rate of appearance (WbRa) for plasma Leu, Phe, Tyr, Arg and urea were calculated from the arterial isotope TTR values for [^13^C]Leu, [^2^H_3_]Leu, [^2^H_5_]Phe, [^2^H_2_]Tyr, [^15^N_2_-^2^H_2_]Arg and [^13^C]Urea, respectively, using the steady-state isotope dilution equation *(1)*:
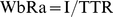
(1)where TTR is the arterial tracer-tracee ratio and Ι is the infusion rate of the tracer. WbRa of [^13^C]Urea reflects total urea synthesis.

#### Splanchnic extraction of Leu (SPELeu)

Splanchnic extraction represents the fraction (as a %) of ingested amino acid taken up by the gut and liver during its first pass, and was calculated as follows:
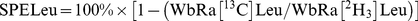
(2)Note that in this calculation, the enrichment of the chosen precursor pool (arterial or venous) falls out of the equation, and thus will not affect the SPELeu calculation.

#### Whole-body protein kinetics

[^2^H_5_]Phe and [^2^H_2_]Tyr were used to assess whole-body protein synthesis (WbPS) and whole-body protein breakdown (WbPB):
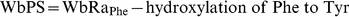
(3)

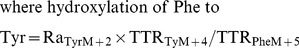
(4)Whole-body Ra of phenylalanine (Ra_end-Phe_) not coming from diet-supplied phenylalanine was calculated as follows:
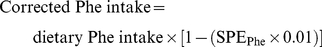
(5)

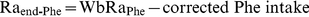
(6)

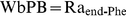
(7)

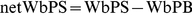
(8)


#### Organ balance tracer measurements

Plasma flow across the portal drained viscera (PDV), liver and hindquarters was calculated using the PAH-based indicator-dilution technique, as described previously [30].

Tissue AA turnover was calculated using a two-compartment model [31]. Fluxes were calculated by multiplying plasma venous-arterial concentration difference by plasma flow. PDV AA fluxes were calculated by multiplying portal venous-arterial concentration difference by mean PDV plasma flow. Splanchnic organ AA flux was calculated by multiplying hepatic vein-arterial concentration difference by hepatic plasma flow. Liver AA fluxes were calculated by subtracting portal drained visceral flux from splanchnic flux. Hindquarter AA fluxes were calculated by multiplying inferior vena cava-arterial concentration differences by mean hindquarter plasma flow.

Fluxes were expressed in nmol/100 g CW/min (where CW is carcass weight) to take into account age-related changes in body composition. A positive flux denotes a net release while a negative flux reflects a net uptake.

Tracer net balance (nb), disposal and production rate (nmol/100 g CW/min) across the PDV, splanchnic region, and hindquarters were calculated for [^2^H_3_]Leu, [^15^N_2_-^2^H_2_]Arg, [^2^H_5_]Phe and [^2^H_2_]Tyr as:
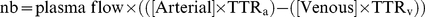
(9)where TTR_a_ and TTR_v_ are the TTR of the measured amino acid in arterial plasma and venous plasma, respectively.

Disposal of an amino acid across the organ was calculated as:
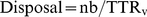
(10)Production of an amino acid across the organ was calculated as
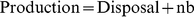
(11)Since Phe degradation in muscle and in portal drained viscera is very low [32], Phe disposal in hindquarters and in portal drained viscera reflects protein synthesis, while Phe production reflects protein breakdown. In the liver, Leu disposal and production represent protein synthesis and breakdown respectively, since Leu degradation in the liver is considered to be low due to low transamination activity [13].

As the rats were studied at the fed state, the total production rate of a substrate measured across the PDV is a combination of the endogenous production of substrate and output of substrate absorbed by the gut from the nutrition infused into the duodenum that was not retained for disposal [33].

Endogenous substrate production in the PDV was calculated as:
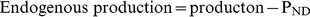
(12)where P_ND_ was derived from the duodenal nutrition infusion (I_NUTR_) and is not metabolized and not disposed of.
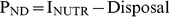
(13)In the results and discussion sections, the portal drained viscera will be referred to as gut and the hindquarter will be referred to as ‘muscles’.

### Statistical analysis

Data are presented as means ± SEM. The Student's *t*-test was used to evaluate differences between adult and old rats in experiment one and between old rats treated with GdCl_3_ or NaCl in experiment two. Statistical analysis was run on “StatView 5.0” software, and the level of significance was set at P<0.05. Between-parameter correlations were analyzed using the Z test.

## Results

### Experiment 1

#### Body composition (table 3)

**Table 3:** Effect of age on body composition.

	Adult		Old	
	g	% of BW	g	% of BW
Body weight (BW)	314±7		670±21+	
Carcass weight	193±4	61.6±0.6	343±8+	51.5±1.1+
Skin+fat weight	77±1	24.5±0.4	217±11+	32.4±0.9+
Intra-abdominal fat	4.6±0.1	1.57±0.04	24.8±4.7+	3.73±0.62*
Liver	9.5±0.4	2.83±0.09	20.2±0.5+	2.95±0.17
Jejunum	3.7±0.3	1.10±0.06	5.3±0.3+	0.78±0.06+
Ileum	4.6±0.1	1.57±0.04	4.8±0.2+	0.71±0.04+
*Gastrocnemius* muscle	1.85±0.06	0.55±0.01	2.11±0.15	0.31±0.02+

Values are expressed as means ± SEM.

*P<0.05 and

+ *P*<0.01 *vs*. adult rats (Student's *t*-test).

BW: total body weight.

Body weight (BW) was higher in old rats. Fat-free mass decreased with age when expressed as a percentage of BW, whereas the fat mass increased. Absolute and relative values of abdominal fat mass were higher and relative *gastrocnemius* mass was lower in old rats than in adults.

#### Liver protein content

Total liver protein content was not significantly affected by age (Adult: 187.6±7.2 *vs* Old: 174.1±10.1 mg proteins/g liver p = 0.3).

#### Gut and liver plasma flows

Gut and liver plasma flows were measured by PAH. There were no significant differences between adult and older rats [PDV Plasma flow group Adult: 10.6±2.5 *vs*, Old: 7.9±2.3 (ml/100 g CW/min) p = 0.3, Liver plasma flow Adult: 12.1±2.1 *vs*, Old: 8.1±2.3 (ml/100 g CW/min) p = 0.1)].

#### Splanchnic extraction of Leu

**Figure 1:**
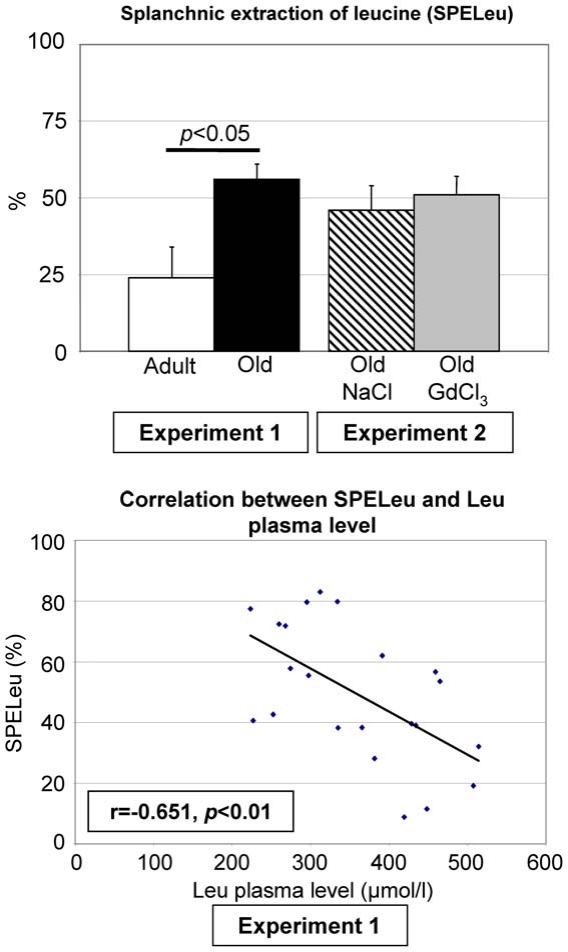
Effect of aging and Kupffer cell invalidation on SPELeu and relationship of SPELeu with plasma Leu levels. Values are expressed as means ± SEM. * *P*<0.05 *vs*. adult group (Student's *t*-test).

[^13^C]Leu Wb rate of appearance (Adult: 2439±298; Old: 875±50 nmol/100 g CW/min) decreased with age (P<0.05), whereas [^2^H_3_]Leu Wb rate of appearance (Adult: 3663±457; Old: 3088±910 nmol/100 g CW/min) remained unchanged. Consequently, first-pass splanchnic extraction of Leu was doubled in old rats (figure 1) compared with adults. This parameter was negatively correlated with net Wb protein synthesis (r = −0.569, P<0.02) and with arterial plasma Leu level (r = −0.651, P<0.01) (figure 1).

#### Arterial plasma concentrations of amino acids (table 4)

**Table 4:** Effect of age on arterial plasma amino acid concentrations at the fed state.

	Arterial amino acid levels						% change from adult to old rats
	µmol/l						
	Adult			Old			
*Essential amino acids*							
Ile	210	±	11	157	±	12+	−25
Leu	435	±	24	319	±	22+	−27
Lys	787	±	43	615	±	43*	−22
Met	164	±	12	98	±	6+	−40
Phe	113	±	7	85	±	3+	−25
Thr	550	±	24	433	±	15+	−21
Trp	56	±	3	41	±	3+	−27
Val	377	±	14	269	±	17+	−29
*Non essential amino acids*							
Ala	1249	±	100	984	±	66+	−21
Arg	274	±	16	200	±	17*	−27
Asn	127	±	5	91	±	4+	−28
Cit	103	±	6	106	±	8	
Gln	1523	±	56	1283	±	50+	−16
Glu	55	±	5	108	±	7+	+96
Gly	806	±	53	704	±	81	
His	166	±	8	126	±	6+	−24
Orn	209	±	18	133	±	8+	−36
Ser	545	±	15	534	±	31	
Tau	277	±	20	271	±	19	
Tyr	76	±	5	65	±	4*	−14
BCAA	1022	±	49	745	±	50+	−27
TAA	8126	±	319	6633	±	239+	−18

Values are expressed as means ± SEM.

*P<0.05 *vs*. and

+ *P*<0.01 *vs*. adult rats (Student's *t*-test). BCAA = branched-chain amino acids; TAA = total amino acids.

Arterial plasma concentrations of AA were lower (20–30% less on average) in old rats compared to adults for all duodenally-infused AA except serine and glycine, for which we did not observe any age-related alteration, and glutamate, which was higher in old rats. Citrulline and taurine were not supplied in the nutrient infusion and their arterial plasma concentrations remained similar between groups.

#### Whole-body protein kinetics (figure 2)

**Figure 2:**
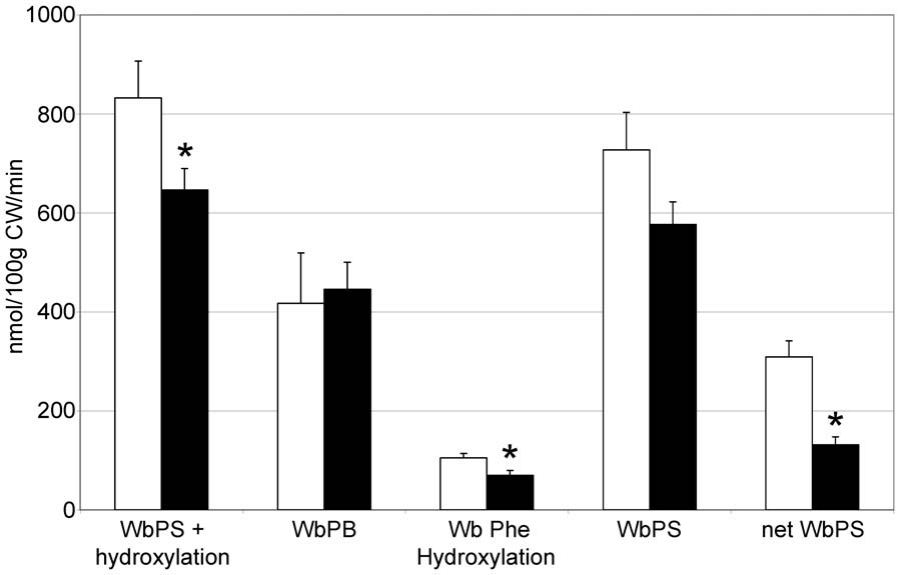
Effect of aging on whole-body protein kinetics at the fed state. □ adult rats and ▪ old rats. Values are expressed as means ± SEM. *P*<0.05 *vs*. adult group (Student's *t*-test). CW = carcass weight. WbPS = whole-body protein synthesis; WbPB = whole-body protein breakdown.

Wb rate of Phe disappearance, which represents Wb protein synthesis plus Wb Phe hydroxylation to Tyr, was lower in old rats than in adults. Wb Phe hydroxylation to Tyr was lower in old rats but Wb protein breakdown and Wb protein synthesis were not different between the two groups. However, net Wb protein synthesis was lower in old rats.

#### Protein synthesis, breakdown and net balance in gut, liver and muscles (table 5)

**Table 5:** Effect of aging on organ protein kinetics at the fed state.

	Synthesisnmol/100 g CW/min		Breakdownnmol/100 g CW/min		Net balancenmol/100 g CW/min	
	Adult	Old	Adult	Old	Adult	Old
**Gut**	345±91	401±59	703±79	575±42	29±7	28±4
**Liver**	1222±511	642±381	1043±415	571±268	48±25	21±12
**Muscle**	107±56	71±23	159±50	98±19	9±4	5±2

Values are expressed as means ± SEM. Groups were compared via a Student's *t*-test. Phe disposal and production in hindquarters and in portal-drained viscera reflects protein synthesis and breakdown in muscle and in gut, respectively. In the liver, Leu disposal and production were used to assess hepatic protein synthesis and breakdown. Cw = carcass weight.

There were no age-related effects on net protein balance, protein synthesis, and protein breakdown rates in the gut, liver and muscles.

#### Urea production and hepatic exchanges of the main ureagenesis precursors (figure 3)

**Figure 3:**
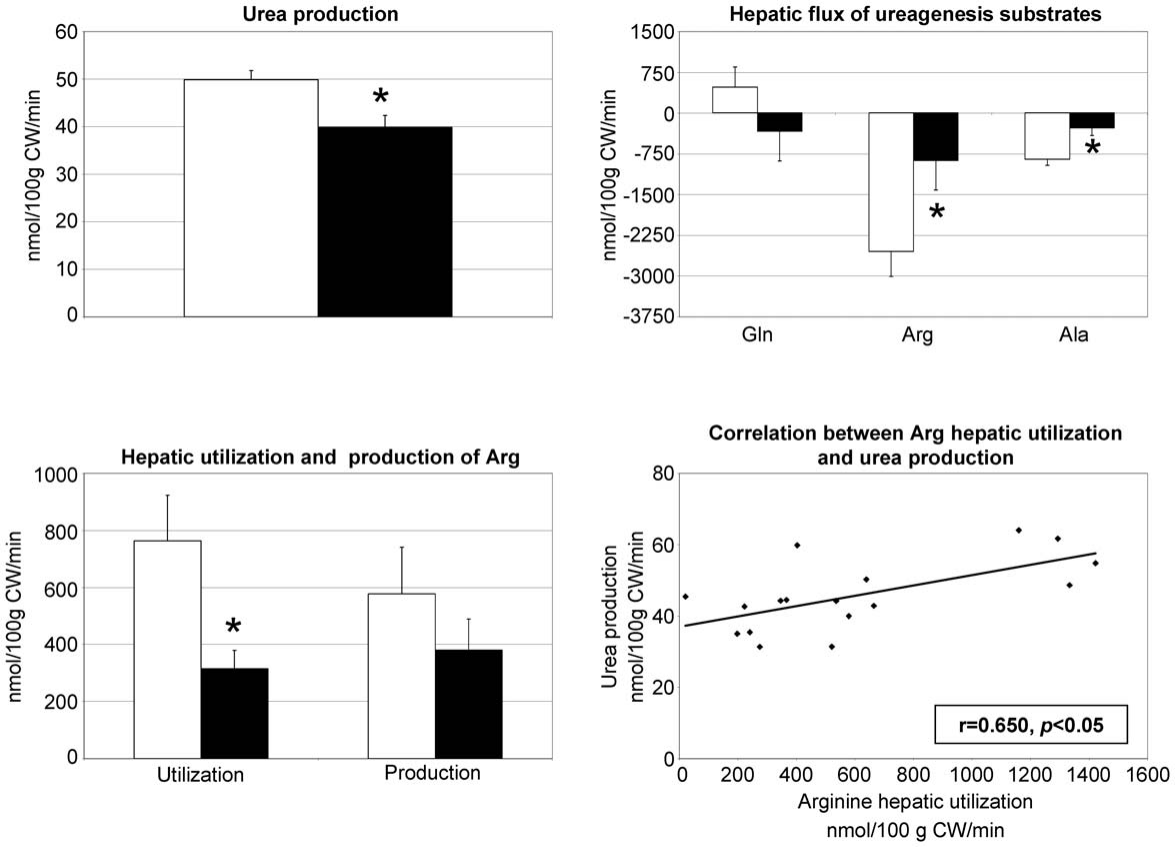
Effect of aging on urea production and hepatic metabolism of major substrates of ureagenesis, i.e. arginine and alanine. CW = carcass weight. □ adult rats and ▪ old rats. Values are expressed as means ± SEM. * *P*<0.05 *vs*. adult group (Student's *t*-test).

Urea synthesis was lower in old rats than in adults. Glutamine (Gln) flux across the liver was not affected by aging. Hepatic uptake of Arg and alanine (Ala) were lower in old rats. Hepatic Arg utilization was lower in old rats, and this parameter was correlated with urea synthesis (r = 0.650, P<0.05) (data not shown).

### Experiment 2

The two groups of old rats presented similar body composition (data not shown).

GdCl3-induced invalidation of KC was unable to normalize SSLeu which was similar between GdCl3- and NaCl-treated rats, and corresponded to the double of the SSLeu recorded for adult rats in experiment 1 (Figure 1).

## Discussion

Our data show that the age-related increase in splanchnic extraction of AA first described in humans [11], [12] also is present in rats. This finding has a dual importance: firstly, as it suggests that age-related SSAA may be a rule in mammals and, secondly, it validates the rat as a suitable model for studying the mechanisms involved in SSAA. The present work was able to rule out a possible implication of age-related increase in ureagenesis, protein synthesis in the gut and liver, and Kupffer cell activation as possible causes of SSAA, and therefore failed to find the primary cause of the SSAA process. Nevertheless, SSAA appears to be of major metabolic relevance in the postprandial state, since this process is correlated with the decrease in net whole-body protein synthesis during feeding and may therefore be responsible for the age-related decrease in lean-body mass.

We observed that splanchnic extraction of Leu was doubled in old rats compared to adults. This result is in agreement with previous results in humans reported by Boirie et al. [11]. Volpi et al. [12] also demonstrated that splanchnic extraction of Phe was higher in elderly subjects than in adults. Our team recently confirmed increased SSAA in healthy elderly for several amino acids, including Leu, Ile, Tyr and Phe but not Val (unpublished data). Therefore, it is generally accepted that an age-related increase in splanchnic extraction is a common feature among ingested AA, and defines the so-called splanchnic sequestration of AA.

We observed that SPELeu is negatively correlated with Leu plasma levels, linking the sequestration of a dietary AA to its lower systemic availability. Thus, the 20–30% lower arterial plasma levels of dietary AA observed in old rats at the fed state might be the consequence of SSAA. Finally, plasma flows were identical regardless of rate age, and cannot therefore explain the change in splanchnic extraction.

The mechanisms underlying SSAA are currently unknown, and constitute the primary focus of the present work. SSAA implies an increased net utilization of ingested AA in the splanchnic area, and we formulated three hypothesis to explain this process 1) an increase in protein synthesis in the gut and/or the liver, 2) an increase in AA oxidation as a cause or a consequence of an increase in urea synthesis, and 3) Kupffer cell activity related to inflammation. A meticulous survey of the literature identified only a handful of studies on the topic of age-related alterations of protein synthesis in the splanchnic area. These studies were conducted in the postabsorptive state and demonstrated that fractional protein synthesis rates in the gut [34] and liver [35], [36] decreased during aging. In the present work, we observed that in the fed state (necessary to study SSAA), protein synthesis in the gut and the liver was not different in old *vs.* adult rats, thereby ruling out an increase in these organs protein synthesis as a potential factor in the age-related enhanced splanchnic amino acid extraction.

In old rats, the results of hepatic protein synthesis could be explained by changes in secretory protein synthesis. Our method measured total liver protein synthesis and thus includes the synthesis of liver secretory proteins like albumin. Therefore, an undetected route of protein synthesized *via* the secretory proteins is unlikely.

It could be hypothesized that liver fat and protein content changes with aging could explain the increased SSAA. However, besides no difference in protein content, we have repeatedly measured liver water content in other experiments but never observed any difference between young and old rats. That said, we have no data on liver glycogen content, which is a limitation of this study. Finally, it is known that protein content in the jejunum is similar between adult and old rats [37].

The splanchnic area is the site of permanent catabolism and irreversible N losses, especially through urea synthesis in the liver [38]. In the postprandial state, this process aims at removing excess nitrogen in order to protect the brain from large amounts of AA [39]. As opposed to our working hypotheses, we observed that urea synthesis is lower in old rats than in adults. One of the major controls of ureagenesis rate is the bioavailability of Arg in the portal vein [40], but we did not observe an age-related effect on Arg portal flux. However, we did observe that old rats had less hepatic uptake of both Arg and alanine (i.e. two major substrates for urea production), and that hepatic Arg utilization was correlated with urea synthesis, thereby demonstrating that the lower hepatic Arg utilization observed in old rats might be participating to the lower urea synthesis. These results are in agreement with our previous work [41] in which we had used an *ex vivo* isolated perfused liver model. The fact that both *in vivo* and *ex vivo* models provide similar results despite using substantially different technologies strongly supports the idea that urea synthesis is impaired in old rats and that an age-related alteration in hepatic Arg and alanine metabolism is involved. In our previous work, we demonstrated that the age-related alteration in alanine uptake was associated with an intra-hepatic accumulation of this AA. In addition, in the present work, plasma glutamate level was doubled in old *vs* young rats. This was a unique behavior among AA and of note glutamate is the sole AA which is produced by the liver [39]. Glutamate plays a role in ureagenesis and is connected with alanine through transamination reactions [39]. This re-enforces the idea that urea cycle is altered with aging. In any case, the fact that urea synthesis decreases during aging demonstrates that sequestrated AA are not over-utilized by this metabolic pathway. From a finalistic standpoint, it is tempting to speculate that the decrease in urea production may be a mechanism established to limit the consequences of splanchnic sequestration of AA and to spare nitrogen in old rats. It would be of interest to trace other key ureogenic amino acids (e.g. arginine and glutamine) in future experiments to gain further insight on aging-related SSAA.

An intriguing feature of the age-related splanchnic sequestration of leucine is that this amino acid is poorly metabolized by hepatocytes which do not possess the enzyme required to initiate the catabolism of BCAA, i.e. BCAA transaminase. Therefore, another cell population in the liver might be involved in the SSAA process. KC are a good candidate, first as the number and basal activity of KC increases with old age in rats, as demonstrated by Hilmer et al. [42], and secondly, as aging is characterized by a low-grade inflammation state [16], [17] which could be responsible for an age-related increase in KC activity. Abdominal adipose tissue (which increases in mass with aging) produces pro-inflammatory cytokines, including TNFα, which in turn activates KC. In response, KC produces interleukin-6, which modifies hepatocyte metabolism and more particularly BCAA metabolism [43]. In addition, as opposed to hepatocytes, KC exhibit BCAA transaminase activity, which is expected to increase when KC are activated [14]. However, the lack of normalization of splanchnic extraction of Leu following gadolinium chloride-triggered KC depletion demonstrates that KC activity is not responsible for SSAA in old rats.

An interesting difference between old and adult rats was the age-related higher amount of intra-abdominal fat (co-localized with splanchnic organs) in the old rats. Several arguments point to a potential role of adipose tissue in the splanchnic sequestration of leucine. First, Boirie *et al*. [11] observed a positive correlation between fat percentage and the splanchnic extraction of dietary leucine. Second, leucine can be transaminated and oxidized in adipose tissue [44], and the rate of leucine oxidation by adipose tissue is not limited by the activity of the branched-chain transaminase [45]. Finally, Lynch et al. [46] showed that leucine promotes protein synthesis in the adipose tissue through activation of the mTOR pathway. However, as we did not measure protein synthesis in intra-abdominal adipose tissue, we can only make the assumption that the increased intra-abdominal fat mass observed in old rats plays some kind of role in the splanchnic sequestration of leucine. This hypothesis warrants further study. A further limitation of our study is that we did not separately address gut and liver contributions to age-related SSAA. Finally, leucine disposal included oxidation, which we did not directly assess.

Even though we have not clearly identified the underlying mechanism of SSAA, we were able to study its effect on peripheral availability of AA and on whole-body protein metabolism. We observed that body composition of old rats underwent a similar pattern of change to that described in elderly human subjects, i.e. an increase in fat mass [47] and a decrease in lean body mass [1], [48]. Old rats showed a decrease in net whole-body protein synthesis, which could contribute to the progressive decrease in lean body mass. Leu is known to be a key anabolic regulator of protein metabolism [49]–[Bibr pone.0027002-Proud1] [51]. We observed a negative correlation between SPELeu and net whole-body protein synthesis, suggesting that the two processes are related. Thus in old rats, SPELeu may contribute to the progressive loss of fat-free mass largely attributed to the loss of muscle mass [8], [52] defining sarcopenia. In the present work, old rats presented a loss of *gastrocnemius* mass normalized for whole-body weight, thus suggesting that they were sarcopenic.

In conclusion, the present study establishes the features of SSAA in rats and rules out an increase in ureagenesis, protein synthesis in the gut and the liver or age-related KC activation as possible causes for SSAA. However, SSAA likely has a major impact on protein metabolism as it most likely participate to the decrease in net whole-body protein synthesis and relative lean body mass observed in old rats, contributing to the decrease in protein accretion characteristic of aging. Further study is needed to determine why dietary AA and especially Leu are sequestrated in the splanchnic area of old rats. Of note, the first step of Leu metabolism (i.e. transamination) is poorly expressed in both the gut and the liver [53], [54]. Based on our data, looking at age-related alterations in Leu aminotransferase activity in these organs could be a promising avenue of research.
